# Central and peripheral nervous system involvement by COVID-19: a systematic review of the pathophysiology, clinical manifestations, neuropathology, neuroimaging, electrophysiology, and cerebrospinal fluid findings

**DOI:** 10.1186/s12879-021-06185-6

**Published:** 2021-06-02

**Authors:** Juan I. Guerrero, Luis A. Barragán, Juan D. Martínez, Juan P. Montoya, Alejandra Peña, Fidel E. Sobrino, Zulma Tovar-Spinoza, Kemel A. Ghotme

**Affiliations:** 1 Translational Neuroscience Research Lab, Faculty of Medicine, Universidad de La Sabana, Autopista Norte, KM 7, Chía, 250001 Colombia; 2 Translational Neuroscience Research Lab, Clinical Neurology Program, Universidad de La Sabana, Autopista Norte, KM 7, Chía, 250001 Colombia; 3 Neurology Unit, Hospital Occidente de Kennedy, Bogota, Colombia; 4 Pediatric Neurosurgery, Pediatric Epilepsy Surgery, Neurosurgical Laser Ablation Program, Upstate University Hospital, 750 East Adams Street, Syracuse, NY 13210 USA; 5 Pediatric Neurosurgery, Department of Neurosurgery, Fundacion Santa Fe de Bogota, Bogota, Colombia

**Keywords:** Central and peripheral nervous system, SARS-CoV-2, COVID-19, Pathophysiology, Clinical manifestations, Neuropathology, Neuroimaging, Electrophysiology, Cerebrospinal fluid findings

## Abstract

**Background:**

SARS-CoV-2 can affect the human brain and other neurological structures. An increasing number of publications report neurological manifestations in patients with COVID-19. However, no studies have comprehensively reviewed the clinical and paraclinical characteristics of the central and peripheral nervous system’s involvement in these patients. This study aimed to describe the features of the central and peripheral nervous system involvement by COVID-19 in terms of pathophysiology, clinical manifestations, neuropathology, neuroimaging, electrophysiology, and cerebrospinal fluid findings.

**Methods:**

We conducted a comprehensive systematic review of all the original studies reporting patients with neurological involvement by COVID-19, from December 2019 to June 2020, without language restriction. We excluded studies with animal subjects, studies not related to the nervous system, and opinion articles. Data analysis combined descriptive measures, frequency measures, central tendency measures, and dispersion measures for all studies reporting neurological conditions and abnormal ancillary tests in patients with confirmed COVID-19.

**Results:**

A total of 143 observational and descriptive studies reported central and peripheral nervous system involvement by COVID-19 in 10,723 patients. Fifty-one studies described pathophysiologic mechanisms of neurological involvement by COVID-19, 119 focused on clinical manifestations, 4 described neuropathology findings, 62 described neuroimaging findings, 28 electrophysiology findings, and 60 studies reported cerebrospinal fluid results. The reviewed studies reflect a significant prevalence of the nervous system’s involvement in patients with COVID-19, ranging from 22.5 to 36.4% among different studies, without mortality rates explicitly associated with neurological involvement by SARS-CoV-2. We thoroughly describe the clinical and paraclinical characteristics of neurological involvement in these patients.

**Conclusions:**

Our evidence synthesis led to a categorical analysis of the central and peripheral neurological involvement by COVID-19 and provided a comprehensive explanation of the reported pathophysiological mechanisms by which SARS-CoV-2 infection may cause neurological impairment. International collaborative efforts and exhaustive neurological registries will enhance the translational knowledge of COVID-19’s central and peripheral neurological involvement and generate therapeutic decision-making strategies.

**Registration:**

This review was registered in PROSPERO 2020 CRD42020193140 Available from: https://www.crd.york.ac.uk/prospero/display_record.php?ID=CRD42020193140

## Background

In 2020, infections by severe acute respiratory syndrome coronavirus 2 (SARS-CoV-2) affected more than 83 million people around the world, and coronavirus disease 2019 (COVID-19) caused more than 1.8 million deaths [1], leading to one of the most devastating pandemics declared by the World Health Organization in the twenty-first Century [2]. SARS-CoV-2 is a 29,903 bp single-stranded RNA encapsulated virus from the Coronaviridae family, betacoronavirus subfamily, capable of affecting the human brain and other structures of the nervous system [2–4].

An increasing number of publications report abnormalities of central and peripheral nervous systems in patients with severe and non-severe COVID-19 [5–8]. Several studies have reported neurological manifestations of COVID-19 and different abnormal findings in ancillary tests. However, no studies in the first months of pandemics have comprehensively reviewed the clinical and paraclinical characteristics of the involvement of the central nervous system (CNS) and peripheral nervous system (PNS) in patients affected by this infectious disease.

The purpose of this systematic review is to describe the characteristics of the central and peripheral nervous system involvement by COVID-19 in terms of pathophysiology, clinical manifestations, neuropathology, neuroimaging, electrophysiology, and cerebrospinal fluid (CSF) findings. This study’s results may help clinicians and researchers approach patients with this condition and generate new inquiries with implications for practice. The explicit questions addressed were: What are the characteristics of the central and peripheral nervous system involvement by COVID-19? What is the described pathophysiology of central and peripheral nervous system involvement by COVID-19? What are the clinical manifestations, neuropathology, neuroimaging, electrophysiology, and cerebrospinal fluid findings in patients with central and peripheral nervous system involvement by COVID-19?

## Methods

We conducted a comprehensive systematic review of all the original studies reporting patients with neurological involvement by COVID-19. We followed the recommendations of the Preferred Reporting Items for Systematic Reviews and Meta-Analyses (PRISMA) Statement [9, 10]. This review was registered in PROSPERO (CRD42020193140) on July 24, 2020.

### Eligibility criteria

We included all original studies, including cohort, case-control, time series, case series, case reports, and letters to the editor containing a complete description of human subjects with confirmed SARS-CoV-2 infection and CNS or PNS involvement. We set the timeframe between December 2019 and June 2020 without language restriction. We excluded studies with animal subjects, studies not related to the nervous system involvement of SARS-CoV-2, publications about coronaviruses other than SARS CoV-2, reports of individuals with suspected or not confirmed infection by SARS-CoV-2, and opinion articles.

### Data source

We conducted a systematic search in PubMed/MEDLINE, Scopus, Cochrane Library, LILACS, and SciELO databases from June 4 to June 30, 2020, using the MeSH terms: (“COVID-19” OR “Coronavirus” OR “Severe Acute Respiratory Syndrome Coronavirus 2”) AND (“Central Nervous System” OR “Peripheral Nervous System”). We added additional terms for amplifying the scope of the review, namely: “CSF”, “Cerebrospinal Fluid”, “Brain AND Spine Imaging”, “Neuropathology”, “Peripheral Neuropathy”, and “Seizures” and sought for individual patient-level data and summary estimates.

### Data extraction

Five reviewers simultaneously screened titles, abstracts, and keywords to check for the fulfillment of inclusion and exclusion criteria. The authors reviewed the resulting articles independently in full text and hand-searched each article’s reference lists to ensure literature saturation. From the resulting studies, we extracted the name of first author, title, year of publication, journal, country, study design, peripheral or central nervous system involvement, and discussed topics (physiopathology, clinical manifestations, neuropathology, neuroradiology, electrophysiology studies, or cerebrospinal fluid findings). We performed a quality assessment at the study level. For case reports and case series, we applied the CARE checklist. Since most of the studies were observational or descriptive, internal validity aspects such as randomization, treatment allocation, or blinding were not applicable. We assessed external validity and generalizability aspects. All reviewers participated in the quality assessment and resolved disagreements by consensus. The data extracted were processed in Excel spreadsheets and categorized into pathophysiology, clinical features, neuropathology, neuroimaging, electrophysiology, and CSF abnormalities.

### Evidence synthesis and data analysis

We performed an evidence synthesis taking into account all selected studies, using a deductive method. Three senior reviewers checked the received data from the selected studies. All authors made decisions and resolved disagreements between individual judgments by consensus. Data analysis combined descriptive measures, frequency measures, central tendency measures, and dispersion measures for all studies reporting neurological conditions and abnormal ancillary tests in patients with confirmed COVID-19.

## Results

**Fig. 1:**
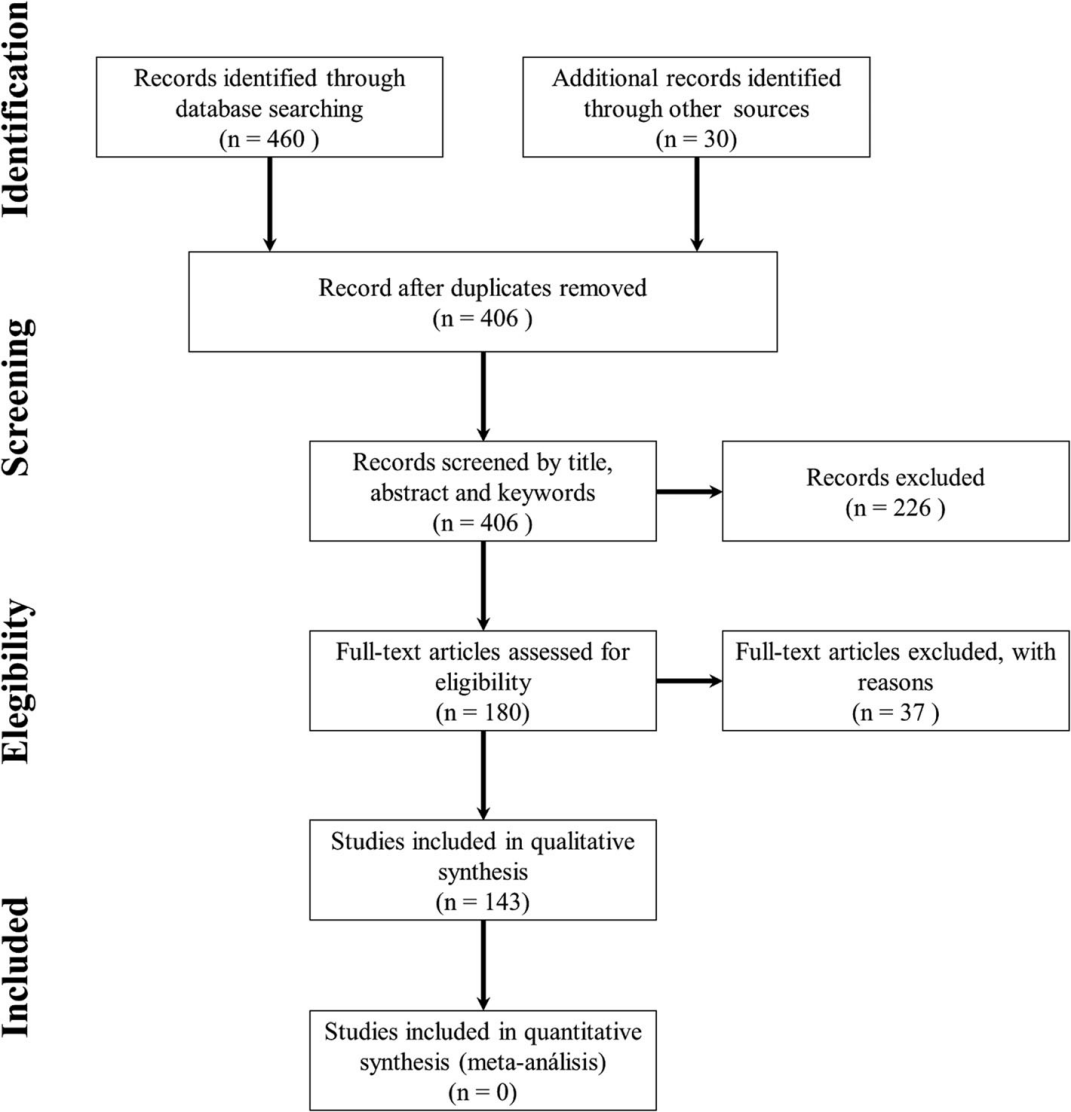
Study selection

This review yielded 143 original publications reporting CNS and PNS involvement by COVID-19, with the selected characteristics alone or combined (Fig. 1).

The included studies were essentially observational and descriptive. From the total, 119 focus on clinical manifestations, 62 describe neuroimaging findings, 60 studies report cerebrospinal fluid results, 51 describe pathophysiologic mechanisms of CNS and PNS involvement by COVID-19, 28 report electrophysiology findings, and four describe neuropathology findings. Among all studies reviewed, we found a total of 10,723 patients with a confirmed diagnosis of COVID-19 who displayed features compatible with neurological involvement. Among them, we found 1633 patients with specific nosological entities or clinical conditions affecting the central nervous system and 43 the peripheral nervous system (Table 1). The remaining 9047 patients reportedly presented with one or more neurological signs and symptoms not attributed to a specific clinical condition or nosological entity. Neurological manifestations of COVID-19 were more common in the inpatient than the outpatient setting (58.5% versus 41.5%, respectively). Indeed, neurological involvement in a patient with confirmed SARS-CoV-2 infection increased the probability of being hospitalized by approximately 81% [11].

**Table 1:** Neurological conditions associated with COVID-19

**Clinical conditions associated with COVID-19 affecting the central nervous system**	**No. of patients / Proportion**
Encephalopathy	990 (60·7%)
Unspecified stroke type	416 (25·5%)
Ischemic stroke	159 (9·7%)
Hemorrhagic stroke	40 (2·4%)
Encephalitis and meningoencephalitis	19 (1·2%)
Acute disseminated encephalomyelitis (ADEM)	4 (0·2%)
Venous sinus thrombosis	3 (0·2%)
Multiple sclerosis exacerbation	2 (0·1%)
Total	1633 (100%)
**Clinical conditions associated with COVID-19 affecting the peripheral nervous system**	**No. of patients / Proportion**
Guillain-Barré syndrome	22 (51·2%)
Other cranial nerve disorders	12 (27·9%)
Facial palsy (Bell syndrome)	5 (11·6%)
Miller-Fisher syndrome and polyneuritis cranialis	4 (9·3%)
Total	43 (100%)

**Fig. 2:**
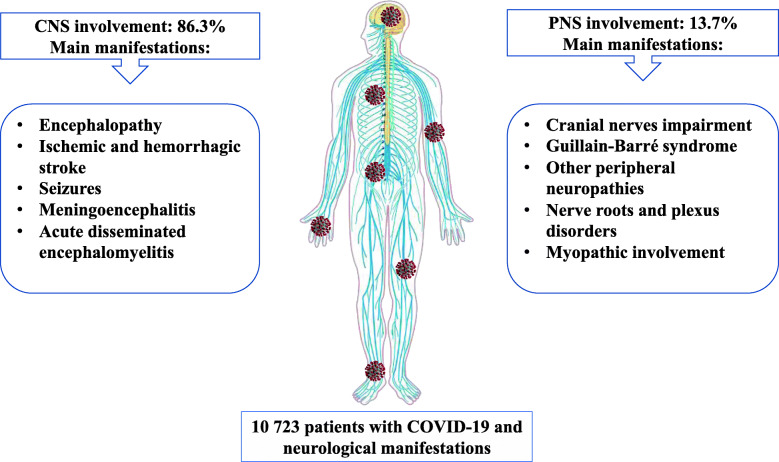
Summary of central and peripheral nervous system involvement by SARS CoV-2. Figure created by the authors using Microsoft PowerPoint partly based on public domain images via Wikimedia Commons. SARS-CoV-2 virus adapted from CDC/ Alissa Eckert, MS; Dan Higgins, MAM, Public domain, via Wikimedia Commons. Nervous system diagram adapted from Medium69, Jmarchn, CC BY-SA 4.0 <https://creativecommons.org/licenses/by-sa/4.0>, via Wikimedia Commons. CNS: Central nervous system; PNS: peripheral nervous system

From the total, 8885 (86,3%) reports of neurological signs and symptoms were related to CNS, while 1414 (13,7%) were related to PNS. Figure 2 summarizes the main neurological manifestations of patients with COVID-19 and CNS or PNS compromise.

Tables 2 and 3 specifically describe the signs and symptoms indicating central and peripheral nervous system involvement, respectively.

**Table 2:** Distribution of signs and symptoms indicating central nervous system involvement in patients with COVID-19

Signs and symptoms indicating central nervous system involvement	No. of patients / Proportion
**Diffuse compromise**	**8129 (91·5%)**
Psychiatric symptoms (including anxiety disorders, mood disorders, psychosis, and insomnia)	4981
Headache	1805
Dizziness	527
Consciousness impairment	416
Delirium	340
Nausea/vomiting	16
Nuchal rigidity	4
Non-specific combination of signs and symptoms	40
**Focal deficit**	**410 (4·6%)**
Extrapyramidal disorders	279
Corticospinal tract impairment	61
Ataxia	18
Dysarthria	13
Amnesia	12
Aphasia	7
Monoparesis	6
Central facial weakness	5
Myoclonus	5
Homonymous hemianopia	4
**Seizures**	**346 (3·9%)**
Non-specified seizures	324
Generalized seizures	9
Non-convulsive status epilepticus	6
Focal seizures	3
Seizure-like events (abnormal involuntary movements)	3
Non-epileptic convulsive syncope	1
**Total patients with CNS signs and symptoms**	**8885 (100%)**

**Table 3:** Distribution of signs and symptoms indicating peripheral nervous system involvement in patients with COVID-19

Signs and symptoms indicating peripheral nervous system involvement	No. of patients / Proportion
**Smell/taste impairment**	**746 (52·8%)**
Anosmia and ageusia	477
Anosmia/hyposmia	128
Ageusia/dysgeusia	141
**Visual impairment**	**9 (0·6%)**
Unspecified decreased visual acuity	8
Complete visual loss	1
**Oculomotor impairment**	**14 (1%)**
Ophthalmoparesis	7
Diplopia	3
Anisocoria	1
Bilateral mydriasis	1
Bilateral abducens palsy	1
Unilateral abducens palsy	1
**Facial palsy**	**13 (0·9%)**
Bilateral weakness/diplegia	7
Unilateral	6
**Other cranial nerve impairment**	**32 (2·3%)**
Glossopharyngeal neuralgia	9
Trigeminal neuralgia	8
Tinnitus	5
Decreased hearing	5
Vasoglossopharyngeal neuralgia	2
Dysphagia	2
Reduced tongue movements/tongue deviation	1
**Peripheral neuropathies involving trunk and limbs**	**353 (24·9%)**
Mixed neuropathy	247
Pure sensitive impairment	31
Paresthesia	30
Hypoesthesia	1
Pure motor impairment	40
Areflexia	14
Distal weakness	8
Tetraparesis	7
Gait difficulties/instability	6
Paraparesis	3
Tetraplegia	1
Paraplegia	1
Neuralgia	8
Limb neuralgia	7
Occipital neuralgia	1
Dysautonomia manifestations	27
**Nerve roots and plexus disorders**	**145 (10·3%)**
**Myopathic involvement**	**102 (7·2%)**
**Total patients with PNS signs and symptoms**	**1414 (100%)**

**Fig. 3:**
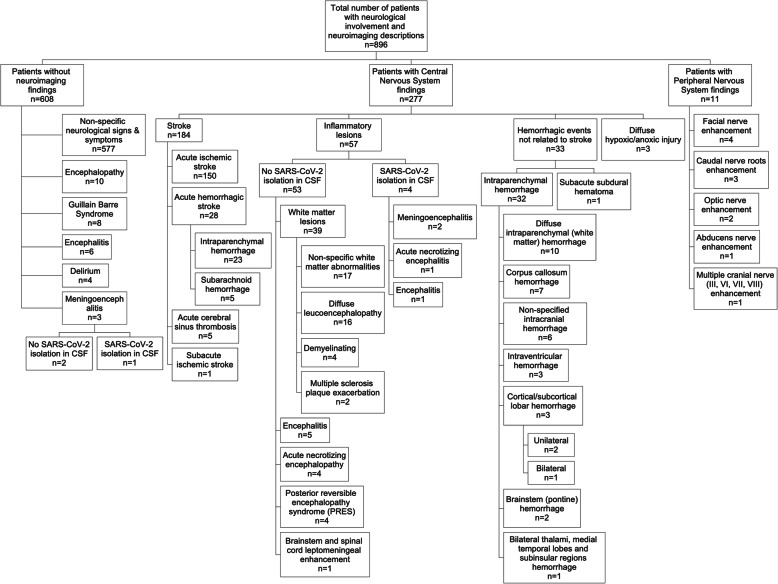
Neuroimaging findings and association with clinical conditions in patients with COVID-19. Figure created by the authors using SmartDraw. CSF: cerebrospinal fluid

Figure 3 summarizes the main neuroimaging findings associated with COVID-19, while Table 4 represents the main CSF findings. Most of the included studies did not report mortality rates explicitly associated with neurological involvement by SARS-CoV-2.

**Table 4:** Cerebrospinal fluid (CSF) findings in patients with COVID-19 and different neurological conditions

Clinical manifestation	N° patients	Proteins (mg/dL)		Cells (cells/μL)		SARS-CoV-2 PCR		
		Average	Range	Average	Range	Positive	Negative	N/M
**Cerebrospinal fluid findings in patients with central nervous system involvement by SARS-CoV-2**								
Encephalopathy	13	67**·**3	0 to 230	9**·**8	0 to 37	0	9	4
Encephalitis	7	83**·**1	19 to 200	40.6	0 to 115	2	5	0
Demyelinating lesions in brain and spine	4	47	32 to 62	1	1	1	1	2
Meningoencephalitis	3	463**·**5	461 to 466	17.3	0 to 21	1	2	0
Stroke	3	50	50	0	0	0	2	1
Seizures	3	66	66	3	1 to 5	0	3	0
Brain vasculopathies	2	292**·**5	78 to 507	6.5	0 to 13	0	2	0
Rhombencephalitis	1	42**·**3	42.3	0	0	0	0	1
Maniac episode	1	190	190	0	0	0	1	0
**Total**	**37**					**4**	**25**	**8**
**Cerebrospinal fluid findings in patients with peripheral nervous system involvement by SARS-CoV-2**								
Guillain-Barré syndrome	17	105**·**9	40 to 193	2.4	0 to 9	0	11	6
Miller-Fisher syndrome	2	75	62 to 80	2.5	0 to 5	0	1	1
Polineuritis cranealis	1	62	62	2	2	0	1	0
Facial palsy	2	44	44	0	0	0	2	0
**Total**	**22**					**0**	**15**	**7**

The reviewed studies reflect a significant prevalence of the nervous system’s involvement in patients with COVID-19. Neurological manifestations appear in a range of 22.5 to 36.4% of all COVID-19 patients among different studies [6, 11–14]. We classified them in diffuse and focal CNS signs and symptoms, seizures, cranial nerve impairment, encephalopathy, neuroinflammatory disorders, acute cerebrovascular disease, and peripheral neuropathies.

### Diffuse CNS signs and symptoms

Fifty-three studies reported 8129 diffuse signs and symptoms of CNS involvement by COVID-19 (Table 2), including neuropsychiatric disorders (61.3%), headache (22.2%), dizziness (6.6%), consciousness impairment (5.2%), delirium (4.3%) nausea/vomiting (0.3%), and nuchal rigidity (0.1%). Psychiatric symptoms included anxiety, mood disorders, psychosis, insomnia, and others. These symptoms are described in depth in other studies [11, 15, 16] and are not the focus of this review. Headache is, indeed, one of the most common neurological manifestations of SARS-CoV-2 infection, with a variability range of 8 to 39% of cases [13, 17]. Headache can be a primary process in these patients or part of a broad spectrum of neurological syndromes such as meningitis, encephalitis, vasculitis, elevated intracranial pressure, and other clinical conditions associated with COVID-19’s neuroinflammatory mechanisms and other underlying systemic causes [7]. Impairment of consciousness and arousal is another common neurological disturbance, documented in up to 37% of patients with COVID-19 as a manifestation of encephalopathy [6, 7]. Delirium was present in 20 to 65% of patients with SARS-CoV-2 infection [18]. It can be attributed directly to SARS-CoV-2 invasive mechanisms to the CNS, leading to a neuroinflammatory response or a multifactorial compromise secondary to sedative therapies, mechanical ventilation, and environmental factors, including social isolation [15]. On the other hand, delirium in critically ill patients with COVID-19 may be a prodromal symptom of infection and hypoxia secondary to severe respiratory failure [15] or an isolated manifestation of COVID-19 [19]. Delirium can also overlap an underlying cognitive impairment, which generates a baseline vulnerability state. However, the elevation of inflammatory markers indicates a concomitant immune response as a precipitant [18]. Furthermore, a history of delirium can increase the risk of post-intensive care syndrome, including cognitive impairment, mental state disorders (such as depression, anxiety, and post-traumatic stress disorder), and physical impairment after leaving the intensive care unit [15]. Dizziness is also a prevalent neurological manifestation, ranging between 7 and 9.4% of patients admitted to intensive care units [20] and 26.21% in general series [6, 17]. Nausea and vomiting are also common neurologic manifestations, with an estimated prevalence of 5% [4]. Their presence may be related to an impairment in the CNS structures related to emesis control in the dorsal vagal complex in the medulla oblongata caused by SARS-CoV-2 [21].

### Focal CNS signs and symptoms

Twenty-eight studies reported 410 patients with focal neurological disturbances (Table 2), including corticospinal and corticobulbar tract impairment, ataxia, dysarthria, amnesia, aphasia, retrochiasmatic visual field alterations, and extrapyramidal disorders. Most of these disturbances were associated with stroke in patients with COVID-19 and are discussed below in this article.

### Seizures

Twenty-eight studies reported seizures in 346 patients with COVID-19. When specified, around 90% of them were new-onset seizures, and 10% occurred in patients with a previous history of controlled epilepsy [22–25]. Most reports did not specify the seizure type in 324 patients, while a few studies documented generalized or focal seizures [24, 26–33], focal or diffuse non-convulsive status epilepticus [34], seizure-like motor events [24], and non-epileptic convulsive syncope [25] (Table 2). Although COVID-19 patients may present seizures due to hypoxia, metabolic derangements, organ failure, or cerebral damage [35], SARS-CoV-2 systemic infection per se represents a minimal risk for seizures during acute illness [36]. In a retrospective multicentric study aiming to evaluate the incidence and risk of acute symptomatic seizures in 304 patients without a prior history of epilepsy, there were no new-onset seizures or status epilepticus during the COVID-19 acute phase [36]. The association between seizures and the severity of COVID-19 remains a matter of debate with evidence in favor [26] and against [22]. There was a previous history of cognitive impairment, older age, and higher levels of creatine-kinase and C-reactive protein after admission for COVID-19 [35] for many patients with seizures. For patients with baseline epilepsy, SARS-CoV-2 infection may trigger seizures; therefore, it is ideal to anticipate breakthrough seizures and prescribe short-term antiseizure medications opportunely [7]. Continuous electroencephalography (EEG) monitoring in any patient with a critical medical condition who has changed in mental status facilitates the timely diagnosis of non-convulsive status epilepticus [14].

### Cranial nerve impairment

Thirty studies reported cranial nerve impairments in patients with COVID-19. Markedly, 746 patients presented smell/taste impairment. Anosmia and dysgeusia/parageusia indicate early involvement of the PNS by SARS-CoV-2, allowing for early screening and isolation of suspected cases before the onset of respiratory symptoms. The neurotrophic properties of SARS-CoV-2 may facilitate access to the CNS through the olfactory nerve and explain why many patients have reported anosmia as a preceding symptom [37]. In COVID-19, the sudden olfactory loss is typically unrelated to nasal swelling or rhinitis [7, 38]. The prevalence of anosmia and ageusia ranges widely from 5% in a study of patients hospitalized in Wuhan to 88% of patients for a cohort study conducted in Germany [7, 12, 38]. Visual deficits reported in COVID-19 include hemianopia in patients with acute ischemic stroke [29, 39, 40] and optic neuritis with acute visual loss [22], associated with optic nerve contrast enhancement in magnetic resonance imaging (MRI). Oculomotor impairment was present in 14 patients, in isolation or as part of a Miller-Fisher syndrome [41, 42]. These patients presented with a compromise of the III, IV, and VI cranial nerves leading to ophthalmoparesis and diplopia. Uni or bilateral abducens’ involvement associated with COVID-19 has been described [42, 43]. MRI studies confirmed nerve enhancement in some of them [44, 45]. Facial nerve compromise by SARS-CoV-2 can occur in isolation [46] or as part of peripheral neuropathy like Guillain-Barré syndrome (GBS) [47]. A group of patients presented with bilateral facial diplegia with unresponsive blink reflex or unilateral facial nerve palsy, around 10 days of SARS-CoV-2 infection [46, 47]. Usual MRI findings in these patients included facial nerve contrast enhancement [44]. Similarly, in some patients with GBS and cranial nerve impairment, III, VI, VII, and VIII contrast enhancement in MRI was evident [45]. Finally, some authors reported compromise of low cranial nerves among patients with COVID-19, including dysphagia as part of GBS [48], isolated dysphagia [49], and hypoglossal deficit due to rhombencephalitis [50].

### Encephalopathy

In this review, 990 patients in 19 studies presented features compatible with acute encephalopathy. Encephalopathy may appear as the predominant disorder at the initial presentation of COVID-19, although most cases rarely progress to severe encephalopathy [30]. Many patients with a clinical diagnosis of encephalopathy had no brain imaging findings. Transmission electron microscopy studies performed postmortem in patients with acute encephalopathy revealed viral particles within cytoplasmic vacuoles of brain capillary endothelial cells in frontal lobe sections. Reverse transcription polymerase chain reaction (RT-PCR) testing of frozen tissue confirmed the presence of SARS-CoV-2 in the brain [51]. The frontal lobe compromise could explain the behavioral changes seen in some patients, and the viral particles in endothelial cells may support a hematogenous dissemination pathway on SARS-CoV-2 into the CNS. Four patients with confirmed COVID-19 presented acute hemorrhagic necrotizing encephalopathy, with findings associated with disrupting mechanisms of the blood-brain barrier that could be related to cytokine storm [28, 52, 53]. Four additional patients presented clinical and imaging features of posterior reversible encephalopathy syndrome (PRES), with acute onset of headache, altered mental status, seizures, and visual disturbances accompanied by fluctuations of blood pressure, with hemorrhagic complications [22, 54]. A multifactorial series of mechanisms related to SARS-CoV-2 infection, along with a breakdown of the blood-brain barrier, may contribute to PRES development in susceptible patients. In our review, EEG findings in several patients with acute encephalopathy included diffuse or focal (frontal or frontotemporal) slow activity wave patterns and some rhythmic discharges [22, 26, 30, 55–58].

### Neuroinflammatory disorders

In our review, 23 patients (in 17 studies) had confirmed CNS inflammatory lesions, including encephalitis, meningoencephalitis, and encephalomyelitis, with variable prevalence [5, 22, 28, 37]. In a cohort of 2660 hospitalized COVID-19 patients, six patients presented with encephalitis as the first and only disorder, two with fatal outcomes [5]. In another cohort of 841 patients, only one patient had confirmed encephalitis [22]. The CSF of patients with inflammatory lesions showed elevated proteins, with an average of 196.3 mg/dl (range of 19–466 mg/dl) and increased cellularity, with 28.95 cells/μL (range 0–115 cell/μL). Most of these patients had normal glucose levels on CSF, although four patients had slightly increased CSF/serum glucose ratio [28, 29, 59]. The isolation of SARS-CoV-2 was possible only in three CSF samples [32, 60, 61]. Several authors reported that CSF cellularity was predominantly lymphocytic [29, 32, 59, 62], reaching 100% lymphocytes in one case [61]. Proinflammatory cytokines in CSF measured in six patients showed high levels of interleukin (IL) 6–8, IP-10, monocyte chemoattractant protein-1 (MCP-1), neurofilament light polypeptide (NFL), glial fibrillary acidic protein (GFAP), tumor necrosis factor-alpha (TNF-α), and B-2 microglobulin [56, 59, 62, 63]. CSF samples of 20 patients with CNS compromise, and four with PNS involvement, ruled out concomitant or alternative infections. Ischemic neuronal damage, demyelination, and viral RNA in and around the hippocampus allowed the confirmation of encephalopathy in a patient who underwent brain biopsy [64]. Other neuropathology findings included perivascular lymphocytes and focal leptomeningeal inflammation. Immunohistochemical analysis showed no cytoplasmic viral staining. Brain sections from five different patients expressed low levels of the virus, but positive tests could be explained by in situ virions or bloodstream viral RNA [65]. EEG studies in patients with neuroinflammatory disorders showed generalized slow-wave activity patterns [61, 62, 66].

### Acute cerebrovascular disease

Twenty studies reported 615 patients with acute stroke. Among COVID-19 patients, the reported prevalence of acute ischemic stroke ranged between 1.1 to 2,5%, increasing to 11 to 31% among those with neurological compromise. The mean time of stroke occurrence was 10 days after the onset of COVID-19 symptoms, although other studies reported an occurrence as early as 2 days after clinical onset [67]. Although the occurrence of cerebrovascular disease induced a two-fold increased risk of a severe form of COVID-19, it did not directly correlate with a significant increase in mortality [68]. Thromboembolic events were the most common neuroimaging finding associated with COVID-19 in this review, including 184 events of acute ischemic strokes. Most of the cases had atypical characteristics, such as multiple arterial territories affected without an identified cardioembolic source, bilateral compromise, a high proportion of vertebrobasilar stroke, arterial dissection, and vasculitis [22, 69, 70]. Most patients presenting COVID-19 associated stroke had baseline cardiovascular risk conditions such as hypertension, diabetes mellitus, hyperlipidemia, smoking, or previous stroke history [13]. In neuropathology findings of selected cases, a few microscopic infarcts were apparent in the neocortex. Hippocampus and cerebellum had scattered necrotic neurons, indicating terminal hypoxic-anoxic injury. Regional infarcts, however, were not present in the brain, brainstem, or spinal cord. T-cells were present surrounding blood vessels and lesions, contrasting with no B cell activation. These non-specific findings can be due to vascular impairment, inflammatory/demyelinating processes, or a combination of mechanisms with direct endothelial cell damage and excessive cytokines release [71]. For hemorrhagic stroke, the estimated prevalence ranged between 0.4 to 2.4%. In this review, 40 patients presented hemorrhagic stroke, 66.5% of which were on anticoagulation therapy [72], and one patient reportedly had a ruptured aneurysm [73]. The fact that SARS-CoV-2 binds explicitly to ACE2 receptors, along with thrombocytopenia observed in severe cases, may lead to an increased risk of a cerebral hemorrhage [13]. Postmortem examinations of selected cases revealed mild brain swelling and foci of white matter hemorrhagic lesions central fibrin with associated extravasated red blood cells, with surrounding reactive gliosis. All lesions presented macrophages at the periphery, axonal damage, and myelin loss. In patients with stroke, CSF studies showed an average protein level of 50 mg/dL; SARS-CoV-2 in CSF was positive in only one case [27, 73]. EEG findings in patients with acute stroke were described only for two patients in one study, one of them demonstrated bilateral slowing of the background rhythm with sharp frontal waves, and the other one showed persistence of sharp slow waves, mainly over the left-hemispheric regions [27].

### Peripheral neuropathies

Fifteen studies reported 22 patients infected with SARS-CoV-2 and GBS, while three studies reported three patients with Miller-Fisher syndrome and one patient with polyneuritis cranialis [41, 42, 44]. The prevalence of GBS accounted for approximately 0.5% of COVID-19 patients, who developed the clinical features between 5 to 10 days after the acute onset of respiratory symptoms [46, 74]. Whittaker et al. suggested that GBS could be a significant neurological sequela of SARS-CoV-2 [12], whereas other authors imply that GBS may occur in patients with COVID-19 without any preceding respiratory or systemic symptoms [48]. Most case reports describe patients presenting with marked lower limb weakness over upper limbs and areflexia that occurred acutely or subacutely. Variable sensory abnormalities have also been described [12]. Nerve conduction and electromyography studies carried out in 16 patients with GBS and COVID-19 reported acute inflammatory demyelinating polyneuropathy in 12 cases [45, 48, 74–81], sensory-motor axonal neuropathy in 3 cases [74, 82], and acute motor axonal neuropathy in 1 case [7]. Three patients with GBS presented enhancement of the caudal nerve roots on spine MRI [44, 74]. Sixteen studies reported CSF findings in 22 patients with SARS-CoV-2-related PNS manifestations. The main findings were albuminocytological dissociation with an average of proteins of 96,6 mg/dL and white cell counts ranging from 0 to 9 cells/μL, without other pathologic findings. SARS-CoV-2 tests were negative in all the CSF samples [41, 42, 45, 46, 74, 75, 79–81]. Concomitant Campylobacter jejuni infection and Lyme disease were also ruled out [48, 77].

## Discussion

To date, this is the most comprehensive systematic review of the central and peripheral involvement of the nervous system by COVID-19 and the only one that integrally assesses the pathophysiology and clinical features, as well as the neuropathology, neuroimaging, electrophysiology, and CSF findings altogether, to analyze their impact in the clinical scenario. Previously reported systematic reviews included a more limited number of articles, ranging between 31 to 37 studies [12, 13]. In many publications, the clinical features and paraclinical findings were siloed and not grouped as nosological entities accounting for the neurological involvement of COVID-19.

In our review, critical neurological events were more frequent among patients with a severe infection than those with mild disease [70]. Patients with COVID-19 admitted with respiratory compromise only, who developed neurological conditions, tended to progress with a more severe course, with increased intubation and mechanical ventilation requirements [83]. Notwithstanding, mortality rates were more associated with the severity of the systemic compromise rather than the neurological involvement.

In most cases in this review, neurological manifestations of COVID-19 resulted from acute nervous system involvement by SARS-CoV-2. Although we cannot establish a causal association between having a history of a neurological condition and increased risk of developing neurological compromise after SARS-CoV-2 infection, in some cases, COVID-19 prompted acute neurological manifestations or exacerbated baseline neurological conditions. For instance, COVID-19 increased the risk for uncontrolled seizures in patients with epilepsy [7, 23, 25, 84], delirium in neuropsychiatric disorders (major neurocognitive disorder, dementia with Lewy-bodies, and schizophrenia) [18], and multiple sclerosis exacerbations [44].

In this review, CNS involvement was more frequent than PNS compromise. Potential explanations include increased brain vulnerability to hypoxia compared to peripheral nerves [85, 86] and a greater expression of ACE2 receptors in the neurons’ soma than in axons and dendrites [87]. The specific mechanisms by which SARS-CoV-2 may affect the nervous system are still debatable. However, there is evidence that combined direct and indirect mechanisms play a role in developing CNS and PNS involvement in patients with COVID-19. This review allowed us to categorize the different pathophysiological processes described below.

### Neurotropic properties of SARS-CoV-2

Specific neurologic manifestations are attributable to the neurotropic mechanisms of SARS-CoV-2, demonstrated by electron microscopy, immunohistochemistry, RT-PCR in brain tissue from autopsy specimens, and CSF tests [2, 3, 32, 51, 88]. Propagation can occur through neuronal dissemination, in which the virus would initially infect the PNS and spread until it gains access to the CNS. Anosmia, a frequent symptom in patients with COVID-19, could be an early indication of this pathway [2]. The mechanisms involved may include retrograde or anterograde neuronal transport through the motor proteins dynein and kinesins [89]. SARS-CoV-2 could gain access through the olfactory bulb and the uncinate fasciculus; it would directly reach the anterior cingulate and the basal forebrain [18, 90]. Brainstem involvement can also occur after exposure to human coronaviruses through the nasal cavity [2, 14].

### Damage to microvasculature

The spike protein S1 enables the virion’s attachment to the angiotensin-converting enzyme 2 (ACE2) receptor, enabling the attachment to the cell membrane [3, 88]. S1 protein in SARS-CoV-2 has 10 to 20 times more affinity for the ACE2 receptor than other coronaviruses. This characteristic might lead to virion’s attachment to the cerebral capillary walls after hematogenous dissemination, followed by a distortion of the blood-brain barrier and viral access to the brain tissue [3, 88]. As the virus gains access to cerebral microcirculation, it can create a hypercoagulability state that may slow the cerebral circulation flow, allowing for increased interaction between the virus and the ACE2 receptors [26]. Glial cells, as well as neurons, express ACE2 receptors; therefore, the attachment of the virus to the receptor could contribute to its neurotropism, which, in turn, could initiate a replication cycle with neural cell damage [3, 88]. SARS-CoV-2 can also cause endothelial damage in the nervous system due to direct infection, activation of the immune system, and thrombo-inflammatory response, leading to microvascular and macrovascular thrombotic events [71, 91]. Given that the glycocalyx covers the entire vascular endothelium, its disruption exposes the endothelial cells to oxidative damage, affecting the microvascular tone and endothelial permeability, maintenance of the oncotic gradient, leukocyte adhesion and migration, and inhibition of intravascular thrombosis [92].

### Brainstem compromise

Animal studies identified the brainstem as a usual target for SARS-CoV. Similarly, cardio-respiratory malfunction in patients with COVID-19 could be due to brainstem impairment by the virus [2, 88, 90]. The brainstem’s compromise can also explain other symptoms such as nausea, loss of appetite, and vomiting in COVID-19 patients. Autonomic nervous system dysregulation relates to brainstem regions such as the solitary tract’s nucleus and the hypothalamus, both parts of the dorsal vagal complex. This abnormality may explain nausea, vomiting, and inappetence described in the disease’s early stages [21].

### Neuroinflammatory response

One way the SAR-CoV-2 can get access to cerebral circulation is by meddling with the immune response. Most of the coronaviruses that affect humans tend to infect the mononuclear phagocytes, leading to the hypothesis that the virus manipulates innate immune response to create a reservoir to access the bloodstream [2, 93]. Endothelial ACE2 receptors’ use to attach to the cell can initiate a coagulation cascade, potentially predisposing patients to microthrombi, acute clots, and stroke [18, 88]. Another mechanism from which SARS-CoV-2 can generate brain damage is by creating a secondary encephalopathy due to inflammation or other systemic viral effects [4, 18], such as cerebral hypoxia or immune dysregulation [15]. Furthermore, SARS-CoV-2 can incite proinflammatory cytokines’ activation in promoting blood-barrier breakdown, especially IL-8 and monocyte chemoattractant protein-1 [26]. Also, the increase in inflammatory markers can create a local cortical irritation that might initiate seizures [26].

### Cytokine storm

SARS-CoV-2 can invade hematopoietic cells and induce a low expression of antiviral cytokines like type I interferon (IFN-αβ) and overexpression of proinflammatory cytokines like TNF-α and IL-6, as well as some chemokines [94]. Besides, a study showed that severe infection by SARS-CoV-2 could create lymphopenia with decreased CD4+ and CD8+, but interestingly, there is no decrease in lymphocytes B. In summary, there is an increase in cytokines IL6, IL2R, IL10, TNFα, and MCP-2 production and a decrease in IFN-γ production [95]. The resulting cytokine storm can create brain dysfunction and prompt neurological symptoms [5]. In other diseases, inflammatory cytokines passing the blood-brain barrier are a causal mechanism of encephalopathy and a significant systemic inflammatory response that could lead to a cytokine storm with a blood-brain barrier breach [18]. Patients with COVID-19 can also have high levels of IL-8 and IL-10 in CSF [59]. This high cytokine concentration in CSF samples may favor the neuroimmunological theory suggested by several authors, which propose that cytokine-mediated damage could be the leading cause of neurological impairment, rather than direct pathogenicity of the virus on the CNS [7, 20, 22, 62, 96].

### Autoimmune response

SARS-CoV-2 spikes interact with the GM1 ganglioside in peripheral nerves, resulting in cross-reactivity and antibody production against these antigens, inducing the peripheral demyelination patterns seen in GBS [97]. Infectious diseases caused by Campylobacter jejuni, Zika virus, and cytomegalovirus show similar molecular mimicry [98]. However, the lack of detection of SARS-CoV-2 in CSF samples may indicate that there is no active intrathecal replication that explains direct demyelination [97]. A systemic inflammatory storm in COVID-19 may also contribute to the amplification of the GBS’s dysimmune process, related to increased blood inflammatory markers (e.g., CRP, IL-6, TNF-α, and IL-1) [99]. On the other hand, neuroinvasion by SARS-CoV-2 may favor leakage of CNS antigenic epitopes (such as aquaporin peptides) to the systemic circulation, leading to autoimmune responses. Although the exact pathogenesis of acute disseminated encephalitis (ADEM) in patients with COVID-19 is still unknown [66, 71], previous research demonstrated cross-activation of antibodies and self-sensibilization of T-cells against myelin related to several bacterial and viral infections, including coronaviruses [100].

### Demyelination

Human coronaviruses that infect the CNS can create a chronic infection and progressive demyelination of the brain, similar to multiple sclerosis [2]. The combination of demyelinating lesions and traces of coronavirus in brain tissue from autopsies and in CSF could explain part of the neuronal compromise of SARS-CoV-2 [101]. SARS-CoV-2 may affect white matter due to various mechanisms, including hypoxia [102], direct injury due to viral neurotropism [102], and autoimmune response combined with endothelial dysfunction [103], which can lead to clinical pictures or imaging compatible with ADEM, neuromyelitis optica spectrum disorder, or multiple sclerosis [66, 71, 104, 105].

### Systemic hypoxia

Severe pneumonia seen in COVID-19 could lead to brain compromise due to hypoxemia caused by the diffuse alveolar damage and inflammatory exudate [106]. These phenomena contribute to vasodilatation, hypercarbia, hypoxia, and anaerobic metabolism, inducing cellular and interstitial edema, low cerebral flow blood, and ischemia [89, 106, 107]. Neuropathology findings support these mechanisms by describing a hypoxic injury to the cerebrum and cerebellum without associated thrombi or vasculitis [65]. Also, SARS-CoV-2 can cause a reduction in dyspnea perception by the indirect toxic effect of cytokines in the corticolimbic circuits and direct effect by affecting ACE2 receptors in cardiorespiratory center neurons in the brainstem [108, 109]. Therefore, COVID-19 may cause a rapidly progressive central respiratory insufficiency, presenting clinically as patients without evident dyspnea despite low blood oxygen levels, aggravating hypoxemia, and prompting early ventilation [87, 108].

Both the central and the peripheral nervous systems may act as a permanent target tissue for neuroinflammatory processes, which has led to theorizing that SARS-CoV-2 infection may trigger or worsen neurodegenerative disorders [94]. COVID-19 infection in children and adolescents may modify long-term cognoscitive capacities and increase the risk of psychiatric disorders because of the cytokine storm effects in the central nervous system [94]. For COVID-19 survivors with specific neurodegenerative disorders such as Alzheimer’s, Parkinson’s disease, multiple sclerosis, and mood disorders, a neuroinflammatory component may play a role in chronic deterioration. Therefore, long-term follow-up of cognoscitive and psychiatric status should be indicated [94].

Very few publications address the therapeutic approach to CNS and PNS involvement in patients with COVID-19. However, there appears to be consensus on the early recognition and timely treatment of these patients. For instance, delirium requires specific and timely therapeutic measures to decrease its impact on the short- and long-term patient’s clinical course [110]. Likewise, the appearance of clinical or subclinical seizures in patients with COVID-19 should prompt starting antiseizure medication since untreated isolated seizures may escalate to non-convulsive status epilepticus, leading to higher morbidity and mortality [35]. Furthermore, a high index of suspicion can lead to early detection and timely treatment of neurological conditions such as stroke, encephalopathy, neuroinflammatory disorders, and peripheral neuropathies.

### Limitations

We acknowledge some limitations in this systematic review, including selection bias since our search algorithm included only publications specifically reporting patients with CNS and PNS involvement. Valuable articles with general descriptions of COVID-19 but not explicitly focused on neurological involvement might contain a significant number of patients with neurological manifestations. Besides, most of the scientific evidence available so far originates in observational and descriptive studies. Although we found a significant number of patients with COVID-19 with neurological manifestations, only a small proportion reported neuroimaging, CSF, electrophysiology, and neuropathology findings. This fact might be related to the scarce but ongoing knowledge generated in scientific publications worldwide as the pandemic progresses. International collaborative efforts and exhaustive neurological registries will enhance the translational knowledge of COVID-19’s CNS and PNS involvement and generate therapeutic decision-making strategies [111, 112].

## Conclusions

This systematic review presents a comprehensive overview of the scientific literature regarding the central and peripheral nervous system’s involvement by COVID-19 and the diverse manifestations in terms of clinical and paraclinical findings. Our synthesis process led to a categorical analysis of the CNS and PNS involvement for all patients included and provided a comprehensive explanation of the reported pathophysiological mechanisms for which SARS-CoV-2 infection may cause neurological impairment. There is a need to conduct prospective cohort studies to analyze the mid and long-term consequences of the CNS and PNS involvement by COVID-19 and randomized clinical trials assessing the efficacy of treatments targeting the neurological compromise by this devastating condition.

## Data Availability

The dataset and raw data supporting the conclusions of this article are available at Intellectum a publicly available, recognized institutional repository owned by Universidad de La Sabana. They can be accessed at http://hdl.handle.net/10818/44055

## Abbreviations

ACE2: Angiotensin-converting enzyme 2
ADEM: Acute disseminated encephalitis
CNS: Central nervous system
COVID-19: Coronavirus disease 2019
CSF: Cerebrospinal fluid
EEG: Electroencephalography
GBS: Guillain-Barré syndrome
GFAP: Glial fibrillary acidic protein
IFN: Interferon
IL: Interleukin
MCP: Monocyte chemoattractant protein
MRI: Magnetic resonance imaging
NFL: Neurofilament light polypeptide
PNS: Peripheral nervous system
PRES: Posterior reversible encephalopathy syndrome
RT-PCR: Reverse transcription polymerase chain reaction
SARS-CoV: Severe acute respiratory syndrome coronavirus
SARS-CoV-2: Severe acute respiratory syndrome coronavirus 2
TNF: Tumor necrosis factor
